# Accurate localization of indoor high similarity scenes using visual slam combined with loop closure detection algorithm

**DOI:** 10.1371/journal.pone.0312358

**Published:** 2024-12-30

**Authors:** Zhuoheng Xiang, Jiaxi Guo, Jin Meng, Xin Meng, Yan Li, Jonghyuk Kim, Shifeng Wang, Bo Lu, Yu Chen

**Affiliations:** 1 Changchun University of Science and Technology, School of Optoelectronic Engineering, Changchun, Jilin, China; 2 China Zhongshan Institute of Changchun University of Science and Technology, Zhongshan, Guangdong, China; 3 School of Computing, Macquarie University, Sydney, Australia; 4 Naif Arab University for Security Sciences, Riyadh, Kingdom of Saudi Arabia; Wilfrid Laurier University, CANADA

## Abstract

Accurate localization is a critical technology for the application of intelligent robots and automation systems in complex indoor environments. Traditional visual SLAM (Simultaneous Localization and Mapping) techniques often face challenges with localization accuracy in high similarity scenes. To address this issue, this paper proposes an improved visual SLAM loop closure detection algorithm that integrates deep learning techniques. Using the TUM f3 loh, Lip6 Indoor, and Bicocca Indoor datasets as experimental bases, a detailed comparison of the proposed algorithm against other methods was conducted across various evaluation metrics. The experimental results show that the proposed loop closure detection algorithm significantly outperforms traditional methods in terms of localization accuracy in high similarity scenes. Specifically, the detection accuracy rates for the TUM f3 loh, Lip6 Indoor, and Bicocca Indoor datasets were 66.67%, 72.72%, and 80.00%, respectively, representing an approximate 18% improvement over the average accuracy of ORB-SLAM2. Additionally, the proposed method demonstrated excellent performance in trajectory error, with a root mean square error (RMSE) of just 0.0816m on the Bicocca Indoor dataset, significantly lower than the 0.1341m RMSE of ORB-SLAM2. Furthermore, improvements in feature extraction and matching mechanisms greatly reduced the occurrence of mismatches, enhancing the system’s adaptability for more accurate localization and navigation in complex indoor environments. The proposed method effectively enhances localization accuracy and system practicality in visually similar indoor environments, offering a new direction for the development of visual SLAM technology and holding significant application potential in intelligent robots and indoor navigation systems.

## 1. Introduction

Simultaneous Localization and Mapping (SLAM) refers to the ability of an intelligent agent to build an environmental model and assess its motion state while navigating an unknown environment using specific sensors [1, 2]. The core of visual SLAM technology lies in accurate self-localization and the construction of environmental maps, forming the foundation and central component of numerous robotic functionalities [3–5] However, in high similarity scenes, such as long corridors or halls with repetitive structures, traditional visual SLAM systems face challenges in recognition and localization accuracy [6, 7]. The high degree of similarity in these environments increases the difficulty of loop closure detection, a critical component of visual SLAM that determines whether the robot has returned to a previously visited location. In visual SLAM systems, loop closure detection compares current visual data with historical information to identify revisited positions, helping to correct localization drift caused by accumulated sensor errors. This technique is essential for improving localization accuracy and system stability. In this study, we focus specifically on highly similar indoor environments, where we improve traditional loop closure detection methods by integrating deep learning techniques to enhance the system’s adaptability to environmental changes and reduce false positives.

Although visual SLAM technology has made significant progress in areas such as dynamic environment detection and spherical image recognition [8–10], its application in high similarity scenes remains a notable challenge. Traditional loop closure detection algorithms face two major issues in these environments. First, the issue of mismatches caused by highly repetitive environmental features. In environments such as long corridors, where visual feature points lack sufficient distinctiveness, traditional loop closure detection algorithms struggle to accurately distinguish spatial positions [11]. Second, the sensitivity of these algorithms to minor visual changes, as even slight variations in lighting or camera angles can impact the stability of feature extraction, thereby reducing the accuracy of loop closure detection [12]. While some researchers have attempted to incorporate deep learning techniques into visual SLAM systems to enhance feature extraction capabilities, solutions targeting high similarity scenes have yet to fully address the issues of mismatches and localization drift.

The primary issue addressed in this study is: how can the traditional loop closure detection algorithm be improved by integrating deep learning techniques to enhance the localization accuracy and robustness of visual SLAM systems in high similarity scenes? To tackle this problem, the study proposes a novel visual SLAM algorithm combined with loop closure detection, specifically designed to address the precise localization challenges in high similarity scenes. Considering that ORB-SLAM2 often suffers from trajectory drift and inaccurate localization due to insufficient feature point matching mechanisms, making it difficult to achieve precise localization in highly similar environments, this research introduces deep learning techniques to enhance the feature recognition and matching capabilities of traditional loop closure detection algorithms. By leveraging convolutional neural networks (CNNs), the system can efficiently learn and extract high-level features from images, significantly improving scene differentiation. The deep learning model also performs complex semantic analysis, reducing the probability of mismatches in visually similar scenes. Additionally, we implemented improved feature extraction and matching mechanisms, combined with real-time performance optimization techniques, to ensure the system’s practicality and efficiency. The contribution of this study lies in the development of a loop closure detection algorithm enhanced with deep learning, which significantly improves the feature extraction and matching capabilities of visual SLAM systems in high similarity scenes. Moreover, this research enhances the localization accuracy of visual SLAM systems in highly similar indoor environments, greatly expanding the potential applications of robots in commercial and industrial settings, especially in spaces with complex structures or repetitive layouts.

## 2. Literature review

### 2.1 Visual SLAM

Since its introduction in 1986, SLAM technology has accumulated nearly four decades of research history [13]. Early developments in SLAM primarily focused on Structure from Motion (SfM) techniques, leading to the emergence of visual SLAM systems such as MonoSLAM, which marked a shift from traditional offline map-building methods to real-time monocular camera localization [14]. Subsequently, the Parallel Tracking and Mapping (PTAM) technology further advanced SLAM by enabling the parallel processing of localization and mapping for the first time [15], a concept that has become central to modern visual SLAM systems. PTAM was also the first system to implement a nonlinear optimization approach, which has had a profound impact on the subsequent development of SLAM technology [16]

With the rise of deep learning technologies, visual SLAM systems began to adopt handcrafted algorithms such as SIFT, SURF, and ORB for feature tracking [17–19]. Among them, the ORB algorithm has been widely used in multiple systems, including ORB-SLAM, due to its fast speed and low memory requirements [20]. However, compared to some deep learning-based local feature algorithms, ORB still lags behind in terms of descriptor discriminability and robustness. Currently, many deep learning-based local feature algorithms have been applied to the front-end of visual SLAM systems. In the visual front-end, feature tracking relies on the camera to capture the surrounding environment. Traditional descriptor-based feature matching methods perform well under significant variations in lighting and texture, but adding descriptors increases computational cost and introduces instability. By combining sparse optical flow tracking with Shi-Tomasi corner detection to replace the use of descriptors, better stability can be achieved in challenging lighting and texture conditions while maintaining low computational cost [21]. Moreover, traditional SLAM modules are increasingly being integrated with deep learning techniques, which is a current trend in research and signals the continued advancement and evolution of visual SLAM technology [22]. The incorporation of deep learning models has significantly enhanced the robustness and real-time performance of SLAM systems in dynamic scenes, demonstrating the great potential of deep learning to improve environmental understanding and scene adaptability in SLAM algorithms [23].

After years of research and advancements, visual SLAM technology has developed a well-established structural framework. A typical visual SLAM system consists of five core components: visual sensors, visual odometry, a back-end optimization module, loop closure detection, and map construction [24]. The primary function of visual sensors is to capture image data for the visual SLAM system, with the main types including monocular cameras, stereo cameras, and RGB-D cameras. Visual odometry, also known as the front-end odometry, calculates the camera’s position by analyzing the transformations between consecutive image frames, thereby tracking the camera’s movement trajectory. The integration of multiple sensor data (such as cameras and LiDAR) can effectively improve SLAM system performance in complex environments. In the future, visual SLAM technology is expected to evolve towards resource-constrained platforms and long-term map building [25].

Visual SLAM methods, due to their low cost, lightweight nature, and rich environmental representation capabilities, are superior to traditional LiDAR-based approaches. However, visual SLAM still faces limitations when dealing with dynamic scenes and challenging lighting conditions [26]. Therefore, it is necessary to further explore the latest fusion strategies in visual-based SLAM algorithms, with a particular emphasis on the potential of combining deep learning with SLAM systems. This study will delve into the application of integrating visual SLAM technology with loop closure detection. The purpose of loop closure detection is to determine whether the camera has revisited the same location within the map and to use this information to optimize pose estimation. As mobile robots move, they must not only perform localization but also use the captured image data and their motion trajectories to construct an environmental map. Currently, visual SLAM is primarily used to build sparse landmark maps.

### 2.2 loop closure detection algorithm

loop closure detection is crucial for visual SLAM systems, especially in the face of complex environments with drastic changes in viewpoints, lighting, and seasons, where it plays a central role in maintaining algorithm robustness. Effective loop closure detection recognizes and confirms whether the camera has passed through a previously visited location, which is crucial to reduce the cumulative error in position estimation [27]. Loop closure detection techniques fall into two main categories: visual bag-of-words-based models and deep learning-based models.

The visual bag-of-words model is implemented by creating a dictionary containing multiple visual words that are extracted from a large number of images and formed by K-means++ clustering. In performing loop closure detection, the system determines whether a loopback has occurred by comparing the similarity between the description vector of the current image and the historical image vector [28]. Although this approach may fail in environments dealing with sparse features or weak textures, it is nevertheless still a traditional technique used in many visual SLAM systems [29].

In recent years, the introduction of deep learning techniques has provided a new perspective on loop closure detection. The concept of deep learning, first proposed by Hinton in 2006, with its unsupervised learning algorithms based on deep confidence networks and layer-by-layer optimization, has been widely used for high-level feature extraction of images [30]. These deep learning-based loop closure detection methods, such as the use of convolutional neural networks and self-encoders, show greater robustness than traditional methods due to their ability to efficiently handle image changes under illumination and viewpoint transformations [31, 32]. However, these techniques require high computational resources, and their application is still challenging in scenes with high real-time requirements or limited resources.

In addition, in order to further improve the efficiency and accuracy of loop closure detection, some researchers have begun to explore methods that fuse traditional image acquisition methods with novel sensing technologies. For example, methods employing RFID, ultra-wideband, and ultrasonic technologies for localization have been proposed one after another. By integrating multiple sensors, these techniques enable robots to accurately detect loopbacks in a given scene, effectively eliminating position drift errors and thus improving localization and navigation accuracy [33, 34]. However, the introduction of more sensors not only increases the complexity of device collaboration and data transmission, but also requires pre-positioning of auxiliary localization devices in the environment, which limits the robot’s ability to operate in unspecifically arranged environments, and in environments with multiple dynamics, the sensor’s localization accuracy is susceptible to factors such as occlusion.

### 2.3 Visual localization methods in complex indoor scenes

Indoor environments are more narrow and variable compared to outdoor ones, and these variations have a large impact at the global level. The lack of texture in most indoor scenes leads to a large concentration of feature retrieval in small regions of the image during visual localization, which in turn affects the robustness of the localization system during the pose optimization phase, requiring multiple iterations. In addition, the fast changing dynamics of indoor environments, as well as the complexity, symmetry, and similarity of indoor buildings, all have an impact on the accuracy and efficiency of matching the 2D features of the image with the 3D point cloud of the scene.

In structure-from-motion (SfM)-based algorithms for generating 3D point clouds for indoor visual localization, high localization accuracy has been achieved by matching local features of 2D images for pre-scene reconstruction and matching local features of the query image with the scene structure during the localization phase [35]. However, the traditional local feature matching method is not capable of coping with large-scale complex indoor scenes, because with the expansion and complexity of the indoor environment, the 3D model becomes larger, which takes more time in matching the query image and tends to increase the proportion of outliers and the iteration time of the RANSAC algorithm, especially in the complex scenes with large viewing angles, wide baselines, and changes in viewpoints, which makes it difficult to realize the efficient and accurate visual localization [36].

By using Gist global features for global search, limiting the search area, and then performing exact matching by detail-rich local features, both accurate and efficient visual localization is achieved, avoiding the high computational cost of global search [37]. On this basis, Redžić et al. (2020) proposed a visual localization strategy based on the plain Bayesian algorithm, which effectively improves the average localization accuracy in indoor scenes when high-precision localization is required through a lightweight threshold fusion method and a position estimation method based on particle filtering [38]. In addition, Lu et al. (2018) improved the way of storing and retrieving image features, and realized fast and accurate localization of query images by storing global features through the constructed KD-Tree [39]. Although partitioning and limiting the image search region can significantly reduce the time cost of localization, it may also reduce the chance of finding the best matching descriptor for the query image, as the excluded feature library region may contain matching feature descriptors, which reduces the localization accuracy. Gopalan et al. (2015) proposed a hierarchical sparse coding method based on geometric priors, which is aimed at avoiding the problem of database partition retrieval that affects the localization effect in complex scenes, learns features useful in location recognition through Grassmann manifolds, achieves sparse coding of image representations, and improves the localization efficiency through hierarchical clustering retrieval [40]. The outlier screening and iterative process in image feature matching also drastically reduces the timeliness of visual localization in scenes with large illumination changes or low light.

## 3. Loop closure detection algorithm design forhigh similarity scenes

### 3.1 loop closure detection process

In the loop closure detection component of visual SLAM systems, the core challenge is accurately estimating the similarity between images. This study utilizes an encoder network to extract feature information from images. By inputting the currently captured image into this encoder, a feature vector is generated. The similarity between this feature vector and those stored in the database is then compared to determine whether the robot has encountered a loop closure.

However, comparing each image with all feature vectors in the database would impose a significant computational burden, which is not only time-consuming but also affects system performance and could potentially disrupt the normal operation of other components within the visual SLAM system. To address this issue, this study proposes a dual-layer similarity assessment structure along with an efficient feature vector storage scheme. This approach ensures accuracy while meeting the system’s real-time requirements. The design of this structure aims to optimize the use of computational resources, thereby enhancing the overall performance and reliability of the system.

#### 3.1.1 loop closure detection framework

The loop closure detection algorithm is a key component in the visual SLAM system, which consists of the following four main steps:

1. Performs pre-processing operations on the captured image data, which includes resizing the image and enhancing the contrast so that the image can be better utilized for subsequent processing.
2. Extract feature vectors from the processed images using the already trained self-coder network and store these vectors with the feature vectors of the historical frames to provide a data base for subsequent comparisons.
3. A preliminary feature matching technique based on the RANSAC algorithm is used to compare the feature vectors of the current frame with those of the historical frames. Based on the quality of matching, the three historical frame feature vectors with the highest similarity are selected for further analysis.
4. An accurate similarity calculation is performed between the current frame and the three selected history frames, and if the calculation result exceeds a preset threshold, it is determined that a loopback has occurred, i.e., the robot re-enters the previous position.

**Fig 1:**
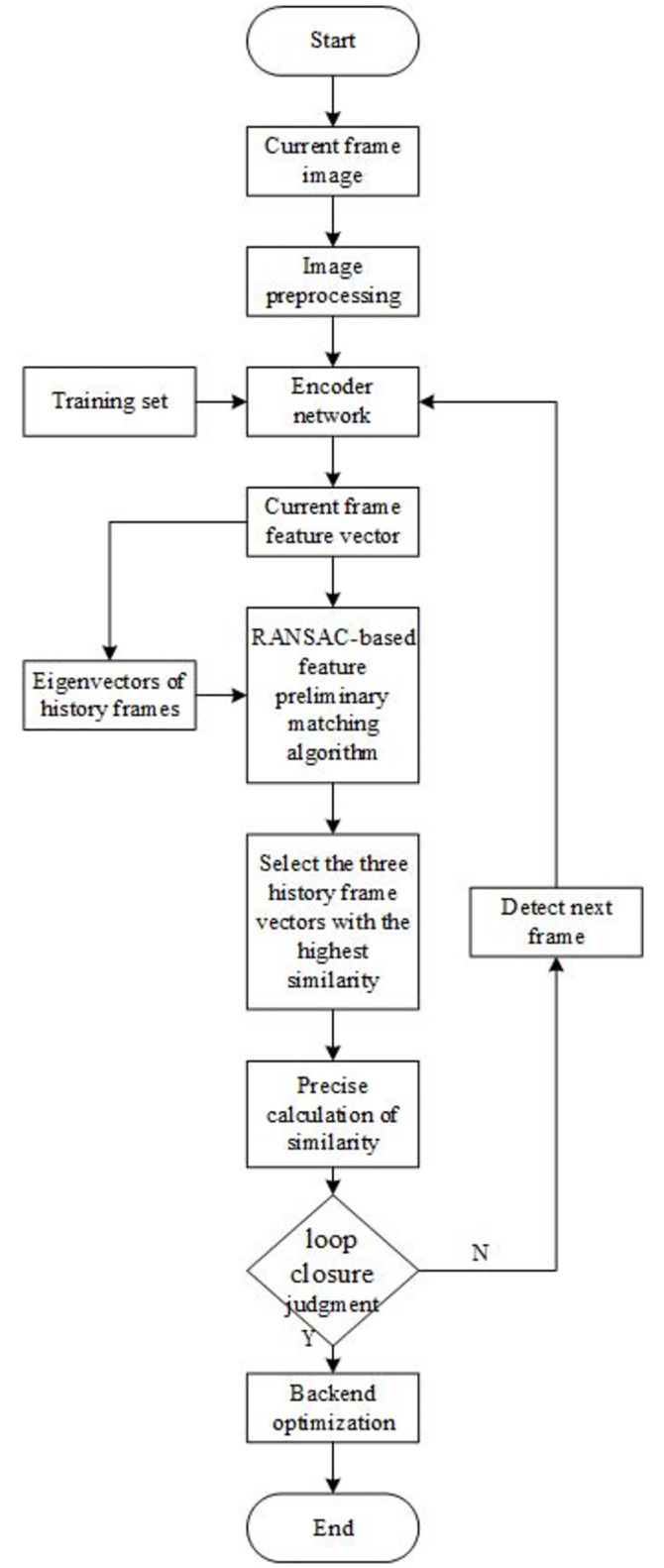
Flowchart of loop closure judgment.

In order to improve the efficiency of the system and prevent processing delays, the third step in the algorithm (preliminary matching of feature vectors) is designed to be executed in a separate thread in parallel with the other steps (image preprocessing, feature extraction, and exact similarity computation). This parallel computing structure effectively avoids data processing bottlenecks and ensures the algorithm’s efficiency and real-time performance. With this strategy, the performance of the visual SLAM system can be substantially improved to ensure its stable operation in dynamic environments. The overall flow of the algorithm is shown in Fig 1.

#### 3.1.2 Image pre-processing

An autoencoder (AE) is an unsupervised neural network commonly used for data compression and feature extraction. Its operation involves two main steps: first, the encoder transforms the input data into a low-dimensional latent space representation, known as a feature vector; then, the decoder attempts to reconstruct the original input data from this feature vector. Through this process, the autoencoder learns efficient data compression representations. In the context of visual SLAM (Simultaneous Localization and Mapping), feature vectors play a crucial role in loop closure detection, primarily used for comparing the similarity between different images. The feature vectors generated by the autoencoder are particularly well-suited for scene recognition and repeated location detection due to their advantages in data compression and key feature extraction. In these applications, cosine similarity is widely used to measure the similarity between feature vectors. Cosine similarity assesses similarity by calculating the angle between vectors, effectively identifying visually similar scenes. An important advantage of using cosine similarity is that its result is unaffected by vector length, focusing solely on the directional relationship between vectors. This is particularly important when evaluating image content similarity, where this method excels in accurately recognizing visually similar images and locations, especially in indoor SLAM scenarios with high visual redundancy. Given that the input to an autoencoder network requires a fixed size, all images in this experiment were uniformly resized to a resolution of 360×480 pixels. This step is essential because images from different datasets vary in size, and standardizing image dimensions helps maintain the consistency and efficiency of the autoencoder network’s processing.

In order to reduce the effect of ambient lighting on the performance of the algorithm and to enhance the recognizability of details and features in the image, this study further applies contrast enhancement to the resized image. In this paper, Histogram Equalization (HE) technique is used. The core of this technique is to redistribute the pixel intensity distribution of the image to achieve a more uniform intensity distribution, thus enhancing the contrast of the entire image. In this way, the lines and element boundaries of the image will become more prominent, thus facilitating more efficient extraction of feature vectors by the self-encoder network. Histogram equalization not only improves the usability of the image, but also provides a more accurate data base for subsequent feature matching and loop closure detection. The main steps are as follows:

Calculate the histogram of the input image and count the frequency of occurrence of each gray level:

$$h(k)=n_{k}$$ (1)

$$P(k)=\frac{n_{k}}{N}$$ (2)

Where *k* represents the gray level and there are *k* ∈ [0,255], *n*_*k*_ is the number of pixels in the image whose gray level is *k N* is the number of pixels in the image.

Calculate the histogram cumulative distribution frequency:

$$S_{k}=\sum_{i=0}^{k}P(k)=\sum_{i=0}^{k}\frac{n_{k}}{N}$$ (3)

Calculate the equalization factor:

$$E_{k}=S_{k}*255$$ (4)

An equalization transformation is performed for each pixel, i.e., all pixels with a gray level of *k* are transformed to pixels with a gray value of *E*_*k*_.

#### 3.1.3 Feature vector extraction with segmented storage structure

**Fig 2:**
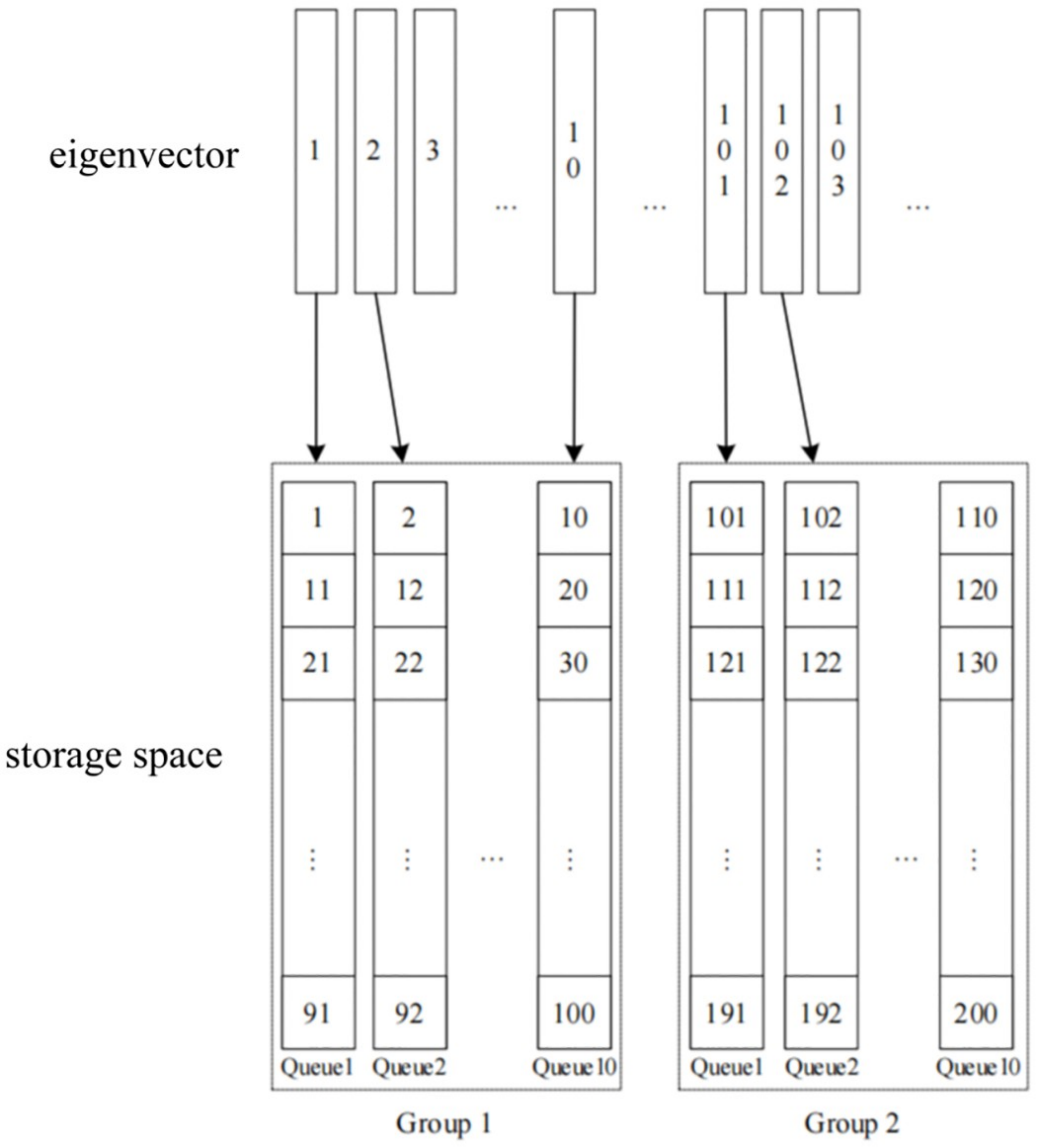
Storage structure of feature vectors.

In this study, an innovative segmented storage structure is proposed for the feature vector storage problem of loop closure detection algorithms, aiming to improve the storage efficiency and optimize the use of computational resources. Compared with the traditional bag-of-words model-based K-fork tree storage method, the storage structure designed in this paper is more suitable to cope with the practical requirements in robot visual SLAM systems (Fig 2).

In the storage structure proposed in this paper, the feature vectors are stored in groups, with each hundred feature vectors constituting a group (Group), and each group is further subdivided into ten queues (Queue), with each queue containing the feature vectors of ten images. The advantage of this design is that it takes into account the high similarity of the scene covered by the ten images taken consecutively by the robot during its actual movement, and therefore, the feature vectors of these images are assigned to the same queue.

The core advantage of this storage method is its efficient data organization structure, which makes it possible to quickly filter out historical data segments that have changed significantly from the current frame scene when performing loop closure detection, and perform a detailed search only within data cohorts or groups that have a high probability of being similar. This not only reduces unnecessary calculations, but also significantly improves the speed and accuracy of loop closure detection. Another benefit that segmented storage brings at the same time is that it avoids the waste of computational resources caused by successive scene detection to a certain extent, as images within the same queue can be assumed to have high similarity without the need to perform a comprehensive loop closure detection for every frame.

#### 3.1.4 RANSAC feature initial matching algorithm

The RANSAC algorithm employs an iterative strategy aimed at estimating the parameters of a mathematical model from a set of observations containing outliers, and is widely used in fields such as data fitting and image processing. The accuracy of the algorithm is improved by increasing the number of iterations, which is essentially a probabilistic algorithm. Its core method is to randomly select a subset of the data from the entire data, use these sample points to estimate the model parameters, and then use the resulting model to validate the other data points. For any data point, if its deviation from the model is within a preset threshold, the point is considered as an in-group; otherwise, it is considered as an out-group.RANSAC repeats the above process for a predetermined number of iterations, and ultimately selects the model with the most in-groups as the optimal estimation. In the application scenario of feature vector matching, the strategy of RANSAC is shown in the pseudo-code, which is detailed in Table 1.

**Table 1:** Preliminary matching algorithm for RANSAC features.

# Initialization of variables L =… # The value for L needs to be specified or obtained from input List = [] # Assuming that the function Y returns a list of 10 vectors and other necessary functions are defined def Y(G, Q). # The specific implementation of this function should return 10 vectors return […] def cosine_similarity(v1, v2). # Calculate the cosine similarity between two vectors dot_product = np.dot(v1, v2) norm_v1 = np.linalg.norm(v1) norm_v2 = np.linalg.norm(v2) return dot_product / (norm_v1 * norm_v2) def find_top_3_vectors(x, vectors). # Calculate the cosine similarity for each vector with x and keep the top three top_vectors = [] for v in vectors: similarity = cosine_similarity(x, v) if similarity > 0.6. top_vectors.append((similarity, v)) top_vectors.sort(key = lambda pair: pair[0], reverse = True) return top_vectors[:3] # Main body of the algorithm for i in range(L): G = random.randint(1, math.ceil(M/100)) # The value of M needs to be defined Q = random.randint(1,10) Y_vectors = Y(G, Q) # Assuming Y returns a list of vectors # Find the top three vectors with the highest similarity to x top_3 = find_top_3_vectors(x, Y_vectors) # Add the results to List (if necessary, this step depends on the purpose of List) List.extend(top_3) # If needed, sort the entire List and extract the final results (this depends on the intended use of List) List.sort(key = lambda pair: pair[0], reverse = True) z1, z2, z3 = [vector for _, vector in List[:3]] # Output print(z1, z2, z3)

The specific steps of the RANSAC feature preliminary matching algorithm are as follows:

From a store with historical frame feature vectors, a specific vector within a group is randomly selected. This selection method is typically used to deal with scenarios that require time series analysis:

$$y_{1},y_{2},y_{3},...,y_{9},y_{10}\in Y$$ (5)

Calculate the difference between the feature vector of the current frame and each of the feature vectors in a certain group, with the purpose of finding the vector with the smallest difference from the features of the current frame. The specific operation is as follows:

$$m=argmin_{j}|x-y_{j}|,j\in [1,10]$$ (6)

where x is the feature vector of the current frame and y_j_ is the history frame feature vector.

Calculate the confidence of matching the minimum difference feature vector with the current frame:

$$S\left(x,y_{m}\right)=\frac{x•y_{m}}{∥x∥∥y_{m}∥}=\frac{\sum_{k=1}^{K}x_{k}\times y_{mk}}{\sqrt{\sum_{k=1}^{K}{\left(x_{k}\right)}^{2}}\times \sqrt{\sum_{k=1}^{K}{\left(y_{mk}\right)}^{2}}}$$ (7)

Where K is the length of the feature vector, the feature vector is *x* = [*x*_1_,*x*_2_,…,*x*_*K*_], *y*_*j*_ = [*y*_*j*1_,*y*_*j*2_,…,*y*_*jK*_]. The matching confidence is *S*(*x*,*y*_*m*_) ∈ [0,1], and its value is close to 1, which represents the higher matching of the image building features.

#### 3.1.5 Precise calculation of similarity

After performing feature matching based on the RANSAC algorithm, this study successfully identified three images of historical frames that matched well with the current frame. The next step is to perform a more accurate similarity analysis of these initial matching results. Given the high demand for accuracy in the processing stage of the algorithm, we adopted the Normalized Cross-Correlation (NCC) method for the accurate calculation of similarity. Normalized cross-correlation is a technique for evaluating the correlation between two vectors or samples of the same dimension, which is particularly applicable to the fields of image processing and pattern recognition.

The normalized inter-correlation method provides a more accurate and reliable similarity assessment by eliminating the effect of signal strength through the normalization process. This method, although computationally expensive, significantly outperforms computational speed considerations in terms of its high accuracy since only a limited number of three historical frame images need to be processed in this study. The steps are as follows.

In order to accurately calculate the similarity between the current frame feature vector *x* and a certain history frame feature vector *y*, the following steps can be followed:

$$\mu_{x}=\frac{1}{K}*\sum_{k=1}^{K}x_{k}$$ (8)

$$\mu_{y}=\frac{1}{K}*\sum_{k=1}^{K}y_{k}$$ (9)

where K is the length of the feature vector *x* = [*x*_1_,*x*_2_,…,*x*_*K*_], *y* = [*y*_1_,*y*_2_,…,*y*_*K*_].

The standard deviation of the eigenvectors is:

$$\sigma_{x}=\sqrt{\frac{1}{K}*\sum_{k=1}^{K}{\left(x_{k}-\mu_{x}\right)}^{2}}$$ (10)

$$\sigma_{y}=\sqrt{\frac{1}{K}*\sum_{k=1}^{K}{\left(y_{k}-\mu_{y}\right)}^{2}}$$ (11)

The covariance of the eigenvectors is:

$$Cov(x,y)=\frac{1}{K}*\sum_{i=1}^{K}(x_{i}-\mu_{x})*(y_{i}-\mu_{y})$$ (12)

The correlation coefficients of the eigenvectors are:

$$NCC(x,y)=\frac{Cov(x,y)}{\sigma_{x}^{*}\sigma_{y}}$$ (13)

The final obtained value of *NCC*(*x*,*y*) represents the similarity between the current frame feature vector x and the history frame feature vector y, and there is *NCC*(*x*,*y*) ∈ [–1,1], the larger the value means the higher the similarity.

### 3.2 Experimental tests

#### 3.2.1 Experimental setting and data set sources

The experiment utilized datasets featuring high similarity scenes, focusing on the TUM f3 loh dataset, Lip6 Indoor dataset, and Bicocca Indoor dataset, which primarily reflect complex office environments. The TUM f3 loh dataset, developed by the Computer visual Group at the Technical University of Munich, is well-suited for various image recognition tasks. Data collection was performed using a high-resolution RGB-D camera equipped with precision motion sensors, capable of simultaneously capturing color and depth images while recording the camera’s exact motion trajectory. This dataset captures highly complex office environments, including repetitive objects and structures such as desks, chairs, and cabinets. The high degree of similarity in objects and layouts in these scenes can easily lead to feature matching confusion in visual SLAM systems, presenting significant challenges for localization accuracy. The Lip6 Indoor dataset primarily includes multiple complex indoor environments, such as corridors, offices, and laboratories. Its high level of spatial similarity and repetitiveness often poses major challenges for visual-based localization methods. Therefore, selecting this dataset is advantageous for validating the accuracy and robustness of the algorithm when dealing with indoor scenes characterized by highly repetitive structures. The Bicocca Indoor dataset, on the other hand, offers a different type of indoor environment, featuring more open spaces and complex geometric structures. This dataset includes large public areas and diverse indoor decorations, which help evaluate the algorithm’s adaptability and performance in dynamic environments. The combination of these datasets, with their varying degrees of scene similarity, provides a robust foundation for testing the improved algorithm’s localization accuracy and robustness in visual SLAM systems.

In the experiment, data recorded using an ASUS Xtion sensor was utilized. The sensor moved along a circular path through multi-textured, multi-structured home and office scenes, forming a loop between the start and end points. The experimental setup included an Intel Core i5-9400F @ 2.90GHz six-core CPU, 16 GB of RAM, and an Nvidia GeForce GTX 1050 Ti 4 GB GPU. The software environment consisted of a 64-bit Ubuntu 16.04 LTS operating system, with CUDA 11.7.1, CUDNN 8.5.0.96, Pytorch 1.13.0, and Python 3.9.

#### 3.2.2 Evaluation indicators and test settings

The experimental evaluation metrics generate the corresponding precision-recall curves from the precision and recall of the algorithms and calculate the area enclosed between the curves and the axes, which are used to comprehensively evaluate the detection effect of the algorithms. Considering that the accuracy of loop closure detection is crucial for camera position correction in visual SLAM systems, wrong loop closure detection (false positives) may lead to serious position errors. Therefore, this study particularly emphasizes the recall rate in the case where the algorithm accuracy is close to 1 as an important evaluation metric.

In order to comprehensively evaluate the effect of the proposed method, the experimental design includes four comparison algorithms: firstly, the ORB-SLAM2 algorithm based on the improved bag-of-words model; secondly, the FLID++ and Dark Net algorithms applying the deep learning technique; and finally, the HOG algorithm verifying the effect of self-encoder neural network feature extraction. In the HOG algorithm, only the encoder feature extraction part of the original algorithm flow is replaced with the HOG descriptor, while the other parts remain unchanged, thus ensuring an accurate comparison of the feature extraction effect in the experiment. This comparison design not only helps to evaluate the efficacy of different algorithms, but also facilitates to reveal the performance of each algorithm in the practical application of visual SLAM systems.

#### 3.2.3 Analysis of results

**Fig 3:**
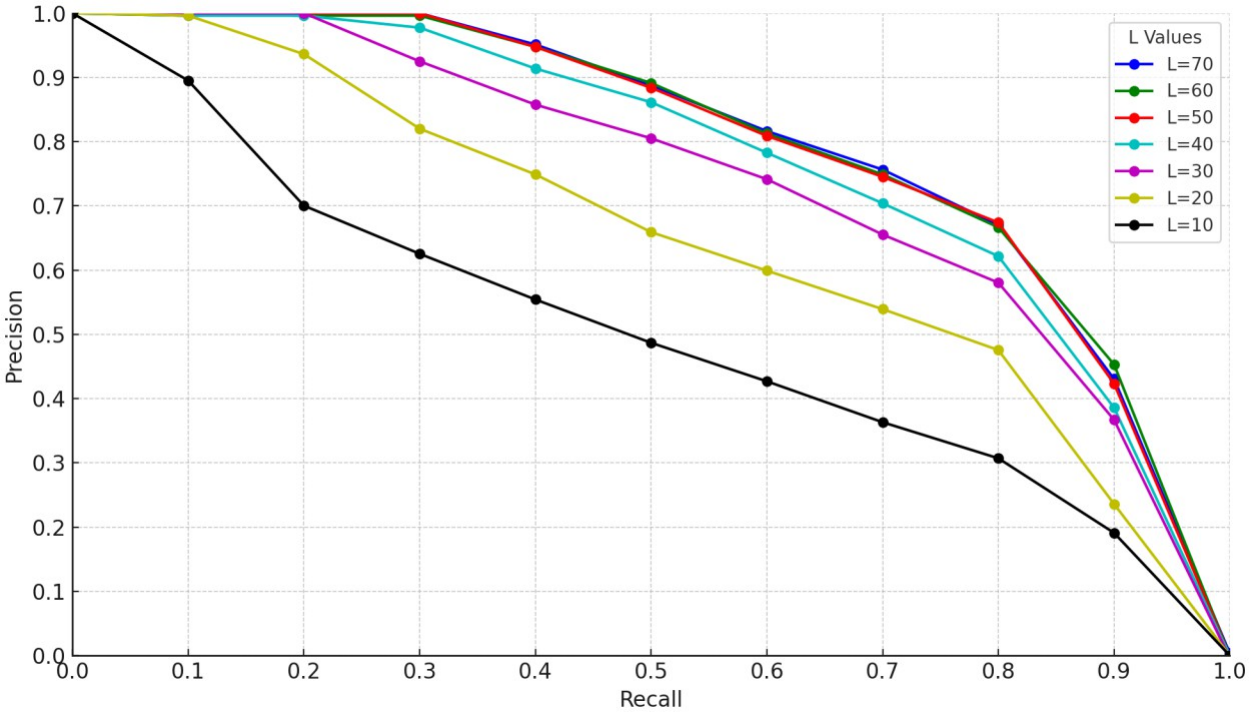
P-R curves of TUM_fr3_loh dataset with different number of iterations.

**Fig 4:**
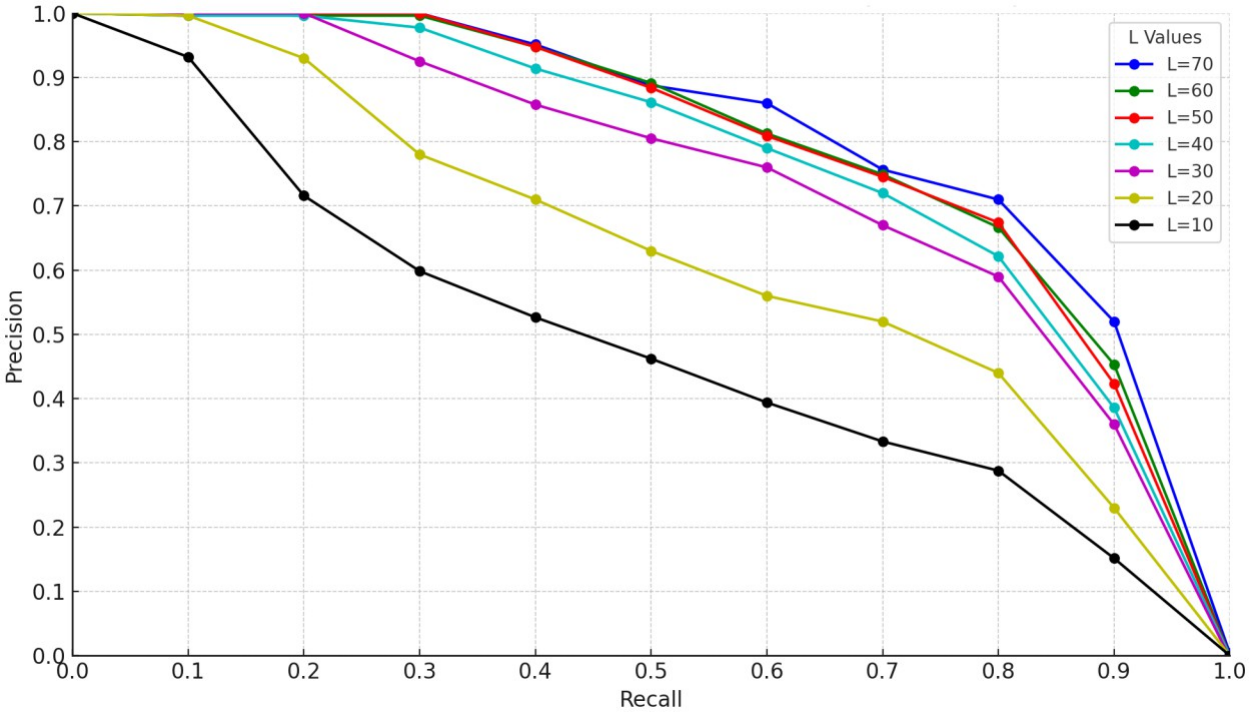
P-R curves of Lip6 Indoor dataset with different number of iterations.

**Fig 5:**
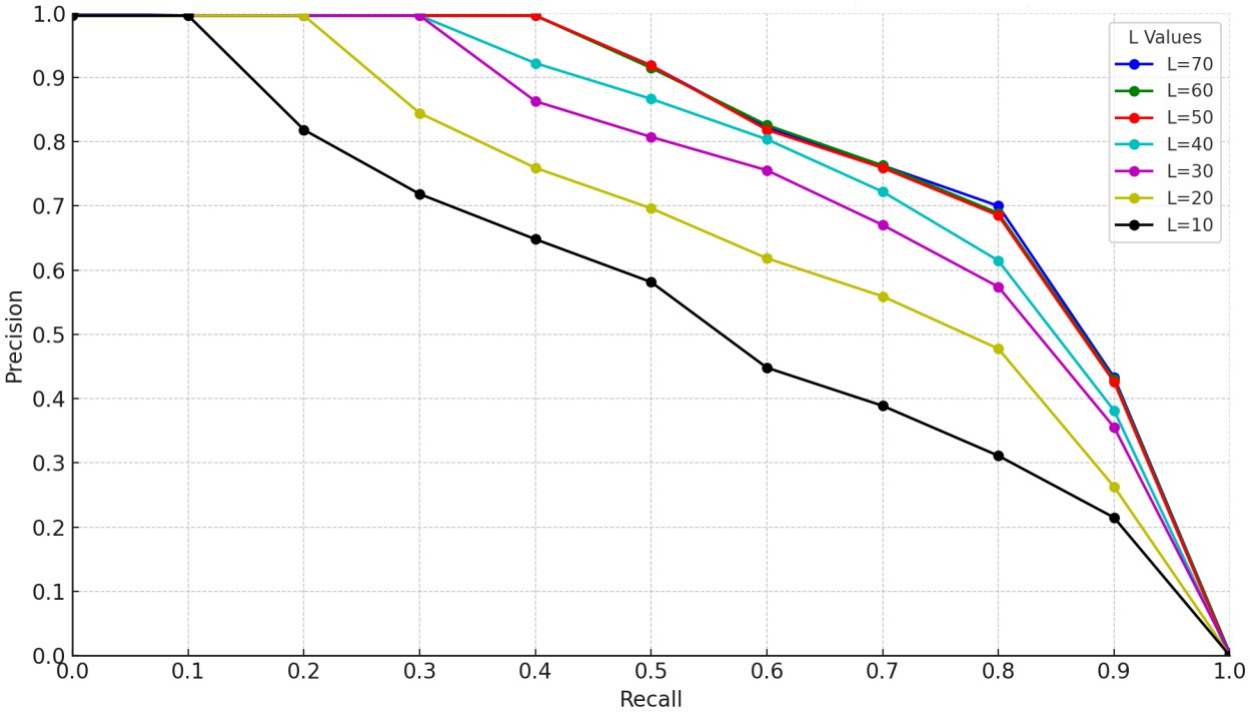
P-R curves of Bicocca Indoor dataset with different number of iterations.

In order to investigate the effect of the number of iterations on the experimental results during the initial matching of features based on the RANSAC algorithm, a series of experiments were designed in this study with multiple rounds of testing by adjusting the number of iterations. These tests aim to determine the optimal number of iterations to achieve the best balance between matching efficiency and accuracy. The experimental results are expressed quantitatively through precision-recall (P-R) curves and area under the curve (AUC), the specific P-R curves are displayed in Figs 3–5 in the text, while the numerical results of AUC are summarized in Table 2.

**Table 2:** Area of the P-R curve sandwiched by the axes at different iteration times.

AUC	10	20	30	40	50	60	70
TUM_fr3_loh	0.5199	0.6720	0.7613	0.7976	0.8221	0.8356	0.8391
Lip6 Indoor	0.4989	0.6483	0.7637	0.7954	0.8321	0.8449	0.8483
Bicocca Indoor	0.5800	0.6939	0.7700	0.8078	0.8334	0.8423	0.8460

**Fig 6:**
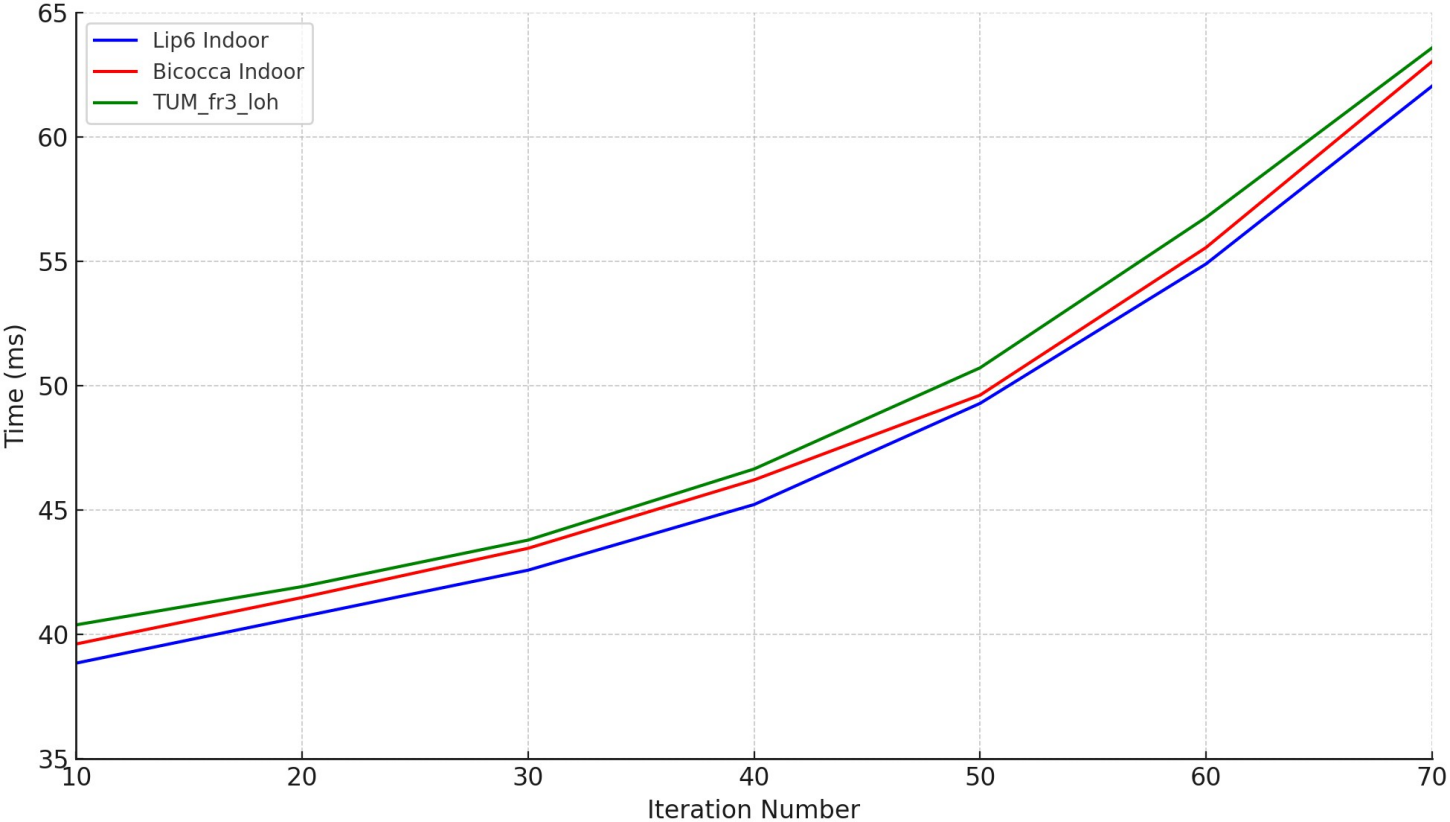
Time variation curves for different number of iterations.

In order to fully evaluate the impact of the number of iterations on the performance of the algorithm, the experiments also recorded in detail the average response time required by the algorithm to process each image frame under different iteration number conditions. These data were counted in detail and presented in Table 3. In order to more intuitively analyze the relationship between the number of iterations and the response time, the experimental results were also used to generate time variation curves, which are presented in Fig 6.

**Table 3:** Comparison of algorithm performance for different number of iterations.

timing	10	20	30	40	50	60	70
TUM_fr3_loh	41.34	42.73	44.49	47.50	51.23	57.71	64.93
Lip6 Indoor	39.82	41.74	43.38	46.13	49.74	55.95	63.56
Bicocca Indoor	40.50	42.36	44.36	47.13	50.52	55.30	64.64

From the analysis of the experimental results, it can be seen that at a smaller number of iterations, the accuracy of the RANSAC-based feature preliminary matching algorithm improves significantly as the number of iterations increases. However, the performance of the algorithm stabilizes when the number of iterations increases to 50, indicating that the algorithm has been effective in finding matching results that satisfy the conditions within the range of no more than 50 iterations. In addition, the curve of the number of iterations versus time consumption illustrated in Fig 6 shows that the increase in time consumption is not linear, but rather exhibits a relatively large increase with the increase in the number of iterations.

Specifically, when the number of iterations exceeds 50, the time cost increases by nearly 11%, despite the fact that the algorithmic accuracy only improves by about 1%. This sharp drop in efficiency suggests that setting the number of iterations to 50 is a more desirable balance in visual SLAM systems. This ensures that the algorithm accuracy meets the system requirements, while also controlling the time cost within a reasonable range and avoiding the waste of resources caused by too many iterations. Therefore, in order to optimize the balance between performance and efficiency, it is recommended that the number of iterations of the RANSAC algorithm be set at 50.

To comprehensively evaluate the performance of our proposed visual SLAM loop closure detection method, we conducted experiments comparing it with six representative algorithms: ORB-SLAM2, SqueezeNet, DarkNet, HOG (Histogram of Oriented Gradients), NetVLAD, and GeM. ORB-SLAM2 is one of the most influential algorithms in the current visual SLAM field, widely used for localization and mapping tasks in various indoor and outdoor environments due to its use of ORB features, which offer high computational efficiency and good real-time performance. Its efficient feature extraction and matching mechanism have made it a classic benchmark algorithm in numerous studies. By selecting ORB-SLAM2 as the comparative benchmark, the performance improvements of the proposed enhanced algorithm, particularly in handling high similarity scenes, can be effectively validated. However, ORB-SLAM2 often encounters feature matching errors in high similarity scenes, leading to a decrease in localization accuracy. This limitation provides an opportunity for the proposed deep learning-enhanced algorithm to demonstrate its superior performance in feature extraction, matching accuracy, and localization precision. Therefore, using ORB-SLAM2 as the benchmark ensures scientific rigor and fairness in comparison, while highlighting the innovation and adaptability of the proposed algorithm in complex environments. SqueezeNet is a lightweight convolutional neural network known for its small model size and high computational efficiency. We included SqueezeNet in the comparison to assess the performance of our algorithm relative to an efficient neural network model in resource-constrained environments. DarkNet, proposed by Guo et al. (2021) is a loop closure detection algorithm based on a deep residual network [41]. It uses an improved triplet loss function to optimize feature extraction, constructing a feature matrix for images and detecting loop closures by calculating cosine similarity. DarkNet was chosen as a comparative algorithm to showcase the application and potential of deep learning techniques in visual SLAM loop closure detection. HOG is a classic image feature descriptor commonly used in object detection. Comparing our method with the HOG algorithm allows us to evaluate the performance differences between traditional feature extraction methods and our deep learning-based approach in complex environments. NetVLAD is a deep learning model specifically designed for image retrieval and loop closure detection. It maps input images into a compact vector space with strong feature representation capabilities, aggregating local features into a global descriptor by simulating the bag-of-words model. By comparing our method with NetVLAD, we can assess the global feature extraction capabilities of our approach in image retrieval and loop closure detection tasks. GeM (Generalized Mean Pooling) is a more advanced pooling method used to extract global features from images. It introduces adjustable parameters on top of traditional max-pooling and average-pooling techniques, enabling the pooling process to adapt to different feature distributions. By including GeM as a comparative algorithm, we can evaluate the ability of our method to aggregate image features in various scenarios and demonstrate its potential for application in complex environments.

**Fig 7:**
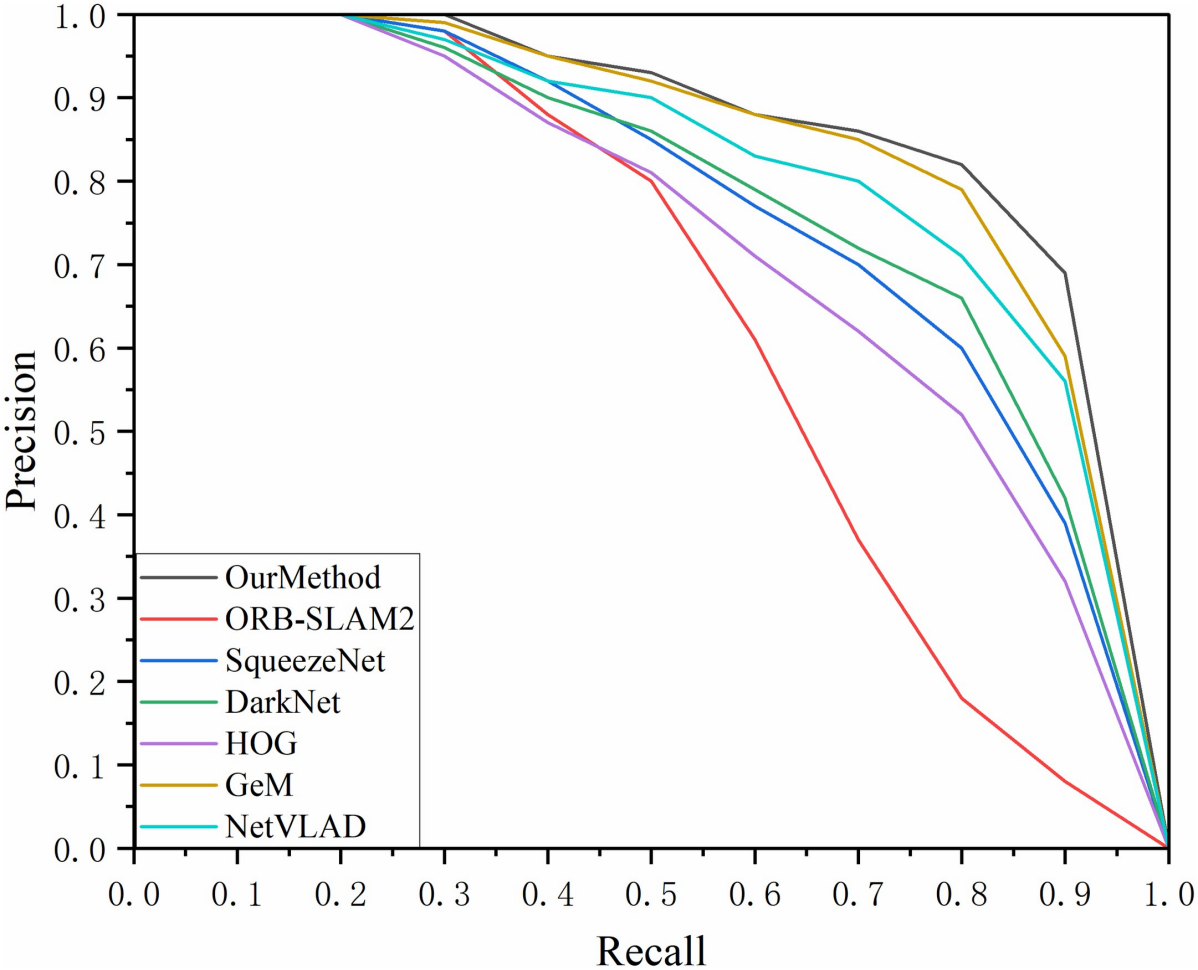
P-R curve of TUM_fr3_loh dataset.

**Fig 8:**
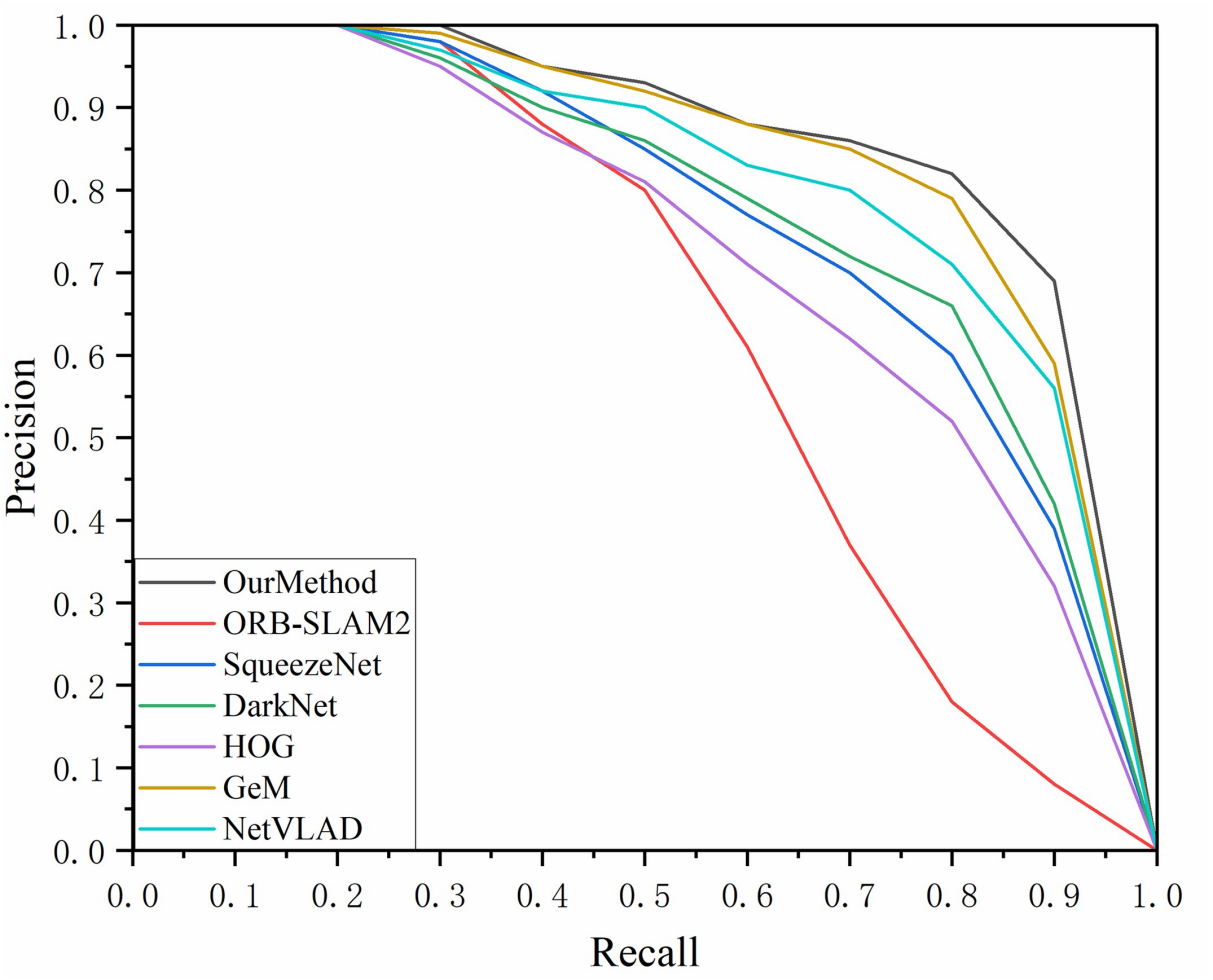
P-R curve for Lip6 Indoor dataset.

**Fig 9:**
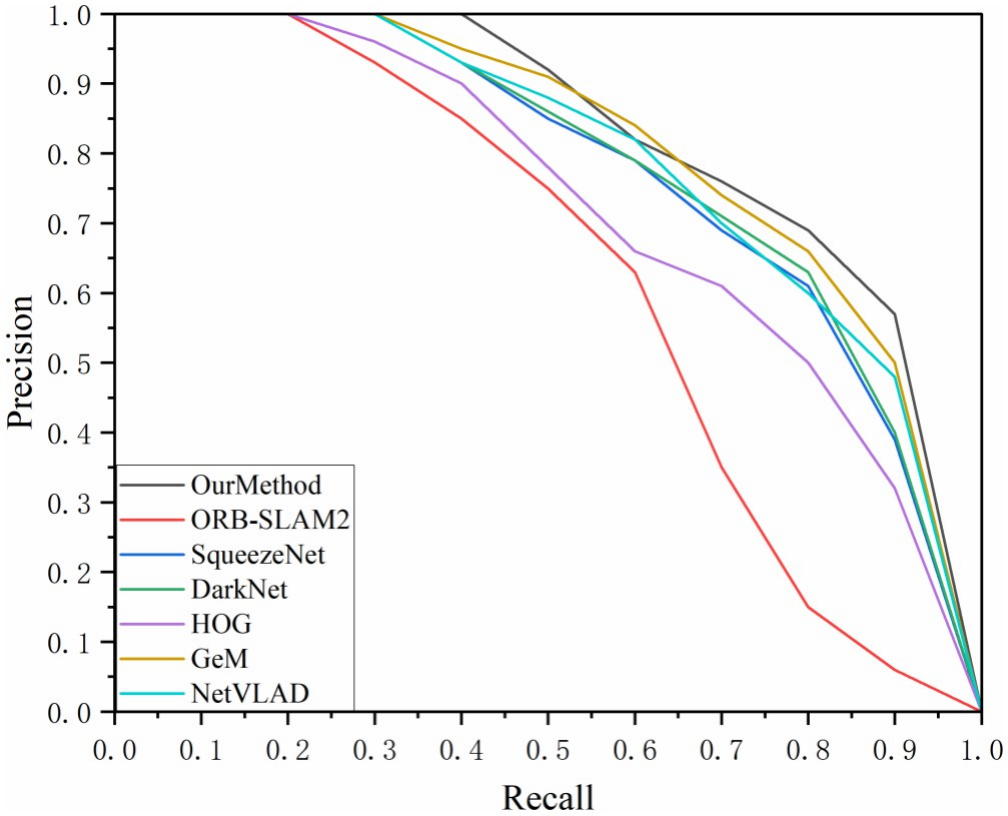
P-R curve for the Bicocca Indoor dataset.

By continuously adjusting the set similarity thresholds, paired data can be obtained for various recall and accuracy rates. This experiment involved three datasets and the results of the P-R (Precision-Recall) curves are shown in Figs 7–9. The area enclosed under the P-R curve and the area formed by the axes were also counted and the results are recorded in Table 4.

**Table 4:** Area of the P-R curve sandwiched between the axes.

AUC	Our Method	ORB-SLAM2	SqueezeNet	Dark Net	HOG	GeM	NetVLAD
TUM_fr3_loh	0.8221	0.6566	0.7911	0.8020	0.7498	0.8103	0.8074
Lip6 Indoor	0.8321	0.6428	0.7988	0.8061	0.7562	0.8142	0.8093
Bicocca Indoor	0.8334	0.6362	0.7993	0.8059	0.7435	0.8195	0.8069

Through comparative analysis, the proposed method consistently achieved an AUC value exceeding 0.83 across all three datasets, outperforming the second-best GeM algorithm by approximately 3% and surpassing the ORB-SLAM2 algorithm by around 28%. Although our method exhibited a lower recall rate compared to the GeM and NetVLAD algorithms when precision dropped to about 0.8 in the TUM_fr3_loh and Lip6 Indoor datasets, the primary focus of this study was to maintain a high recall rate while ensuring high precision. In multi-class algorithm testing, our method maintained 100% precision at a recall rate of 35%. Even at a 70% recall rate, the accuracy of our method remained above 0.8 in the Lip6 Indoor and Bicocca Indoor datasets, and it stayed above 0.75 in the TUM_fr3_loh dataset, demonstrating greater stability and accuracy compared to other algorithms. Additionally, the autoencoder-based feature extraction network designed in this study exhibited superior performance compared to algorithms utilizing traditional HOG descriptors. This advantage can be attributed to the fact that, despite using HOG descriptors for comparison training, our method employed a randomized projection transformation algorithm for image preprocessing. This preprocessing step enhanced the network’s adaptability to slight changes in perspective, enabling it to effectively extract consistent feature vectors even in the presence of minor image jitter—an aspect that is particularly crucial for loop closure detection tasks.

By comparing the performance of the algorithms on the three datasets longitudinally, it can be observed that the method proposed in this study performs better on the BicoccaIndoor and Lip6 Indoor datasets, with an AUC value of more than 0.81. Comparatively, the algorithm’s performance on the TUM f3 loh dataset is slightly less impressive. This phenomenon may stem from the fact that the environment of the TUM G3 loh dataset is predominantly an office scene, which includes complex desktop layouts with items such as computers, flower pots, and bookshelves, which may interfere with the algorithm’s recognition ability. In contrast, the BicoccaIndoor and Lip6 Indoor datasets mainly contain scenes in the category of corridor passages, and although the similarity between the scenes is high, their overall geometric elements are relatively simple, making feature extraction relatively easy, which facilitates the algorithm’s performance.

In addition to comparing the accuracy profiles of the algorithms on the three datasets, the experiments also statistically evaluated the temporal performance by counting the average response time of each algorithm for each image, and the statistical results are shown in Table 5.

**Table 5:** Algorithm performance comparison (ms/frame).

	Our Method	ORB-SLAM2	SqueezeNet	Dark Net	HOG	GeM	NetVLAD
TUM_fr3_loh	51.23	39.98	56.56	48.28	46.13	50.26	51.33
Lip6 Indoor	49.74	38.56	52.53	46.11	44.49	50.01	55.69
Bicocca Indoor	50.52	40.10	55.05	48.77	45.39	52.17	53.41

In visual SLAM systems, a processing speed exceeding 10 frames per second is required, meaning that the processing time per output image should be less than 100 milliseconds per frame. According to the data presented in Table 5, the proposed algorithm achieves a processing time of 49 milliseconds per frame, which clearly meets the real-time requirements of visual SLAM systems. While ORB-SLAM2 demonstrated the best performance in terms of speed, our method’s processing time increased by 25% compared to ORB-SLAM2. However, this increase in processing time is accompanied by a 28% improvement in detection accuracy. Compared to other deep neural network-based algorithms, although the processing time is similar, our algorithm demonstrates a significant advantage in detection accuracy. This confirms the effectiveness of the proposed method, providing a favorable balance between computational efficiency and detection performance.

## 4. Implementation of SLAM accurate inspection system for indoor visual

### 4.1 System framework and system architecture

Suppose the robot carrying the camera moves from time *t* = *k*-1 to t = *k* and its position changes from *x*_*k*-1_ to *x*_*k*_. During this process, the robot captures images of the surrounding environment through the camera *y*_*k*-1_, *y*_*k*_ to obtain environmental information. The visual SLAM problem can be represented as:

$$x_{k}=f(x_{k-1},y_{k-1},y_{k})$$ (14)

This expression performs the solution of the current position *x*_*k*_ by means of the position *x*_*k*−1_ and the observation *y*_*k*−1_, *y*_*k*_. Where *f* is a function that describes the visual SLAM process.

The visual SLAM system includes image sensors, front-end visual odometry, back-end optimization algorithms, loop closure detection module and map building module. The image sensor is responsible for capturing the visual information in the environment and providing raw data for the system. Based on these data, the front-end visual odometry processes the image, extracts key features, and determines the camera position through feature matching and motion estimation, a process that is key to positioning accuracy. The map building module transforms the data obtained from the front-end computation into a 3D scene map in real time, while the loop closure detection module monitors the similarity between the current frame and the historical frames to determine whether the robot returns to a previously visited position. Once a loopback occurs, the back-end optimization algorithm steps in to adjust and optimize the accuracy of the entire map.

**Fig 10:**
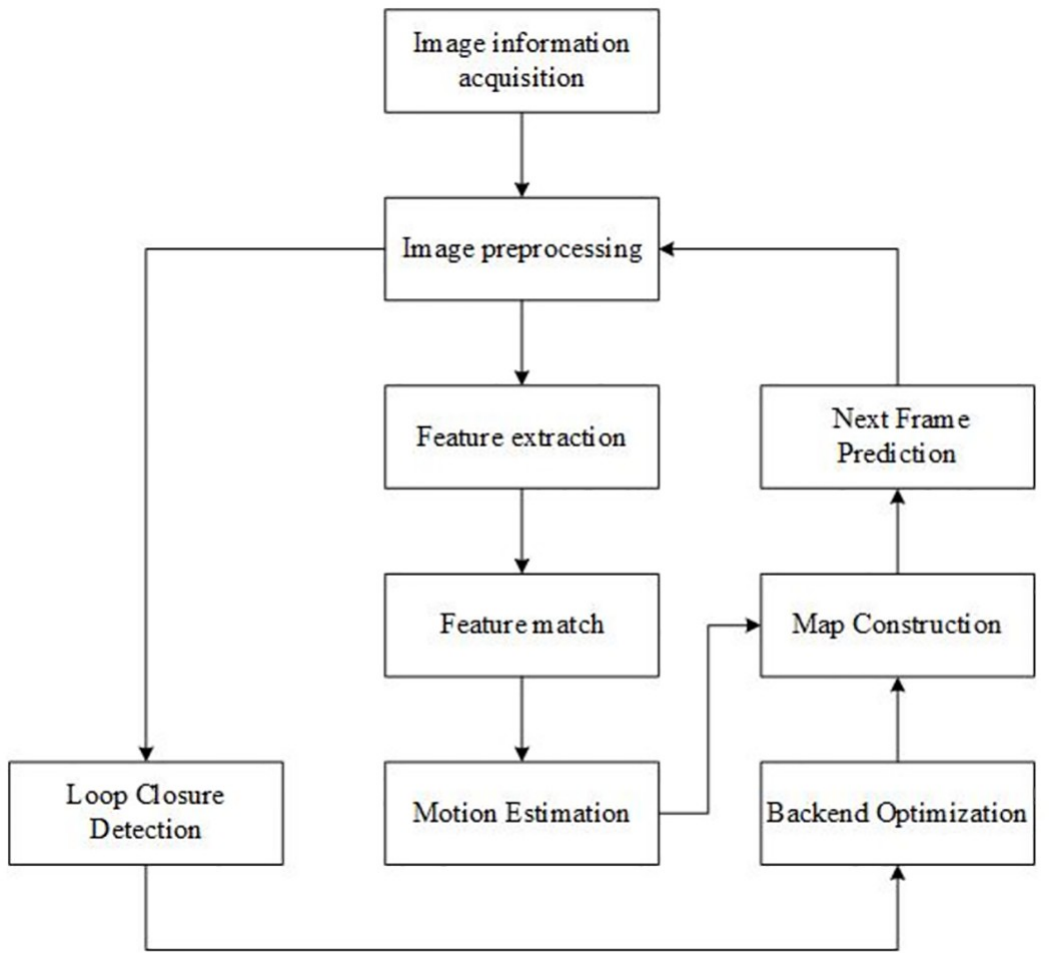
Overall framework of visual SLAM system.

In addition, the system is designed with efficient utilization of computational resources in mind by dividing the key processing tasks into three parallel threads-tracking thread, map-building thread, and loopback thread, as shown in Fig 10. Each thread focuses on its specific task, ensuring that the entire system works together and operates efficiently.

**Fig 11:**
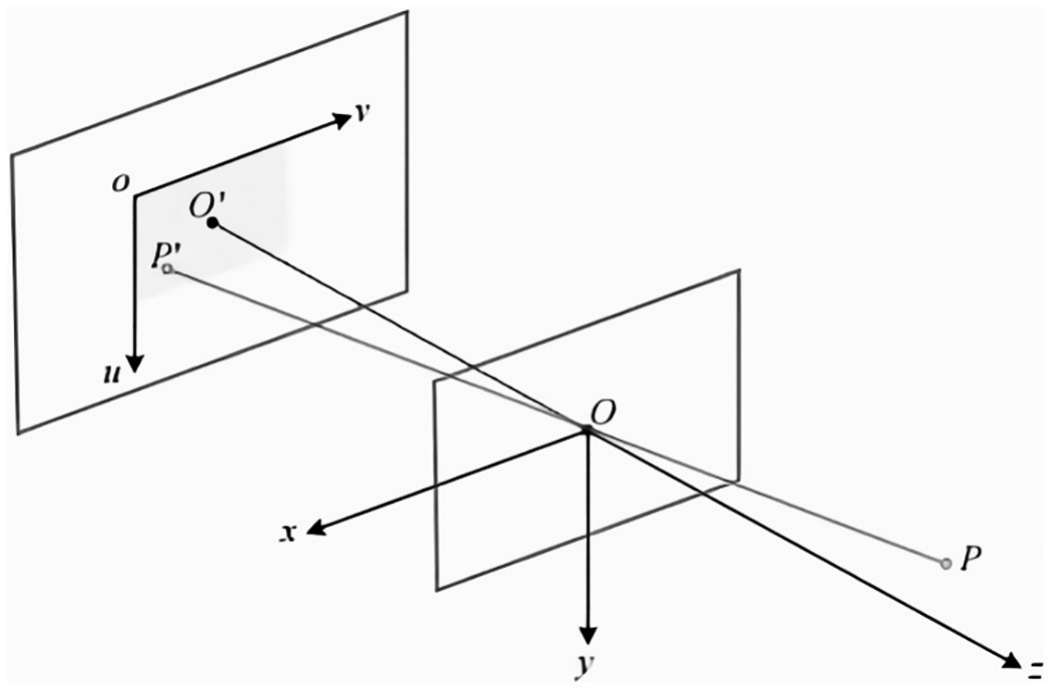
Pinhole camera model.

The imaging principle of the RGB camera used in the visual SLAM system can be described by means of the pinhole model, which is described and illustrated in detail in Fig 11. The pinhole model essentially utilizes the principle of small-hole imaging, where an inverted image is formed by passing through a pinhole followed by an inverted image as a way of simulating how the camera captures and processes light, and thus estimating the robot’s position and attitude in space.

In Fig 11,*O* − *x* − *y* − *z* is called the camera coordinate system, and the optical center point of the camera is set as *O*, any point in the three-dimensional space *P* through the optical center of the projection point on the imaging plane of the camera is *P*′, after the pinhole projection, the optical center displayed on the imaging plane of the camera *O* is set as *O*′, and the distance between *OO*′ is the focal length of the camera *f*.

In the camera coordinate system, the coordinates of the point *P* are [*X*,*Y*,*Z*]^*T*^ and the coordinates of the point *P*′ are [*X*′,*Y*′,*Z*′]^*T*^. According to the geometrical relationship of the imaging of the small hole, the spatial relationship between the two points can be obtained:

$$\left\{\begin{array}{l} X^{\prime }=f\frac{X}{Z}\\ Y^{\prime }=f\frac{Y}{Z} \end{array}\right.$$ (15)

In order to convert the projected image into pixel coordinates, we need to use a pixel coordinate system on the imaging plane *o* − *u* − *v*, where the upper left corner of the image serves as the coordinate origin, the u-axis is parallel to the x-axis and positive to the right, and the v-axis is parallel to the y-axis and positive downward.

Let the pixel coordinate of *P*′ be [*u*,*v*]^*T*^, the transformation between the pixel coordinate system and the camera coordinate system includes a scaling and translation process, so the spatial coordinate of the point *P*′ can be expressed as:

$$\left\{\begin{array}{c} u=\alpha X^{\prime }+c_{x}\\ v=\beta Y^{\prime }+c_{y} \end{array}\right.$$ (16)

where *α* and *β* are the scaling multiples on the x-axis and v-axis, respectively, and *c*_*x*_,*c*_*y*_ is the translation distance of the origin o on the corresponding axis.

Let *f*_*x*_ = *αf*, *f*_*y*_ = *βf*, and then expressed in the form of a join matrix, we have:

$$Z\left[\begin{array}{c} u\\ \nu \\ 1 \end{array}\right]=\left[\begin{array}{ccc} f_{x} & 0 & c_{x}\\ 0 & f_{y} & c_{y}\\ 0 & 0 & 1 \end{array}\right]\left[\begin{array}{c} X\\ Y\\ Z \end{array}\right]≜KP$$ (17)

Eqs (5) and (6) depicts the transformation relationship between the pixel coordinates of the image obtained by the camera at a point in three-dimensional space and the camera coordinates. Where ***K*** is referred to as the internal reference matrix of the camera, the establishment of ***K***, the process of camera calibration, mainly involves the accurate determination of the internal parameters such as focal length, principal point position and pixel distortion. The Zhang calibration method used in this study is done with the help of Matlab software and OpenCV library and includes the following key steps:

1. A tessellated calibration board consisting of two-dimensional squares is photographed from several different angles;
2. Calculate the single-stressor matrix using the corner points on the checkerboard grid;
3. Calculate the inner and outer reference matrices of the camera based on these matrices;
4. Apply the least squares method to estimate the distortion factor;
5. Minimizing reprojection error for global parameter fine-tuning.

Once calibration is complete, the images captured by the camera undergo initial pre-processing such as scale unification and contrast enhancement, which are necessary steps before the images are fed into the visual odometer for feature extraction.

Visual odometry is a key technology that utilizes camera image sequences to estimate the relative position and attitude of a robot. Its core principle is to calculate the camera’s motion trajectory by analyzing the changes of feature points in consecutive image frames, and to obtain the camera’s integrated motion path by integration method. In the practice of visual odometry, it is mainly divided into two methods: feature point method and direct method. The feature point method focuses on extracting salient feature points from an image and determining the displacement and rotation of the camera through feature matching with subsequent motion estimation. The visual SLAM system implemented in this paper adopts the feature-point method, which consists of key aspects such as feature extraction, feature matching and motion estimation, aiming at accurately tracking and estimating the camera’s motion in complex environments through these successive processing steps.

The feature extraction session is a key step in visual SLAM, involving the identification of key points with discriminative ability from images that are essential for subsequent feature matching and motion estimation. In this process, the robustness, speed, and accuracy of the algorithms for detecting, describing, and screening feature points must be considered comprehensively, and the technique that best suits the specific application scenario and the available computational resources must be selected. In this study, the ORB feature descriptor is selected because it possesses scale invariance and rotation invariance, is robust to illumination, noise and viewpoint changes, and has fast computational speed, which is suitable for fast matching between consecutive frames. The specific realization steps include:

1. Image pre-processing: before feature extraction, the image is processed with denoising, scale normalization and contrast enhancement to improve the effect and accuracy of subsequent feature extraction;
2. Feature point detection: the ORB algorithm is applied to detect feature points from the processed image.
3. Feature point description: the detected feature points are described, and each feature point is represented by a vector to represent its local features, which facilitates the subsequent matching process.
4. Feature point filtering: Filter the feature points with the best differentiation and stability from the detected feature points to speed up the matching speed and reduce the unnecessary computation.

The feature matching session, on the other hand, searches for the corresponding feature points in the two images to determine the relative positional transformations between the two images. The main steps are divided into two parts: feature point matching and matching point screening. The former matches the feature points in the two images by calculating the Euclidean distance between the ORB feature descriptors. The latter screens the matching points based on geometric consistency and descriptor distances to exclude the mis-matched points and reduce the computational burden.

**Fig 12:**
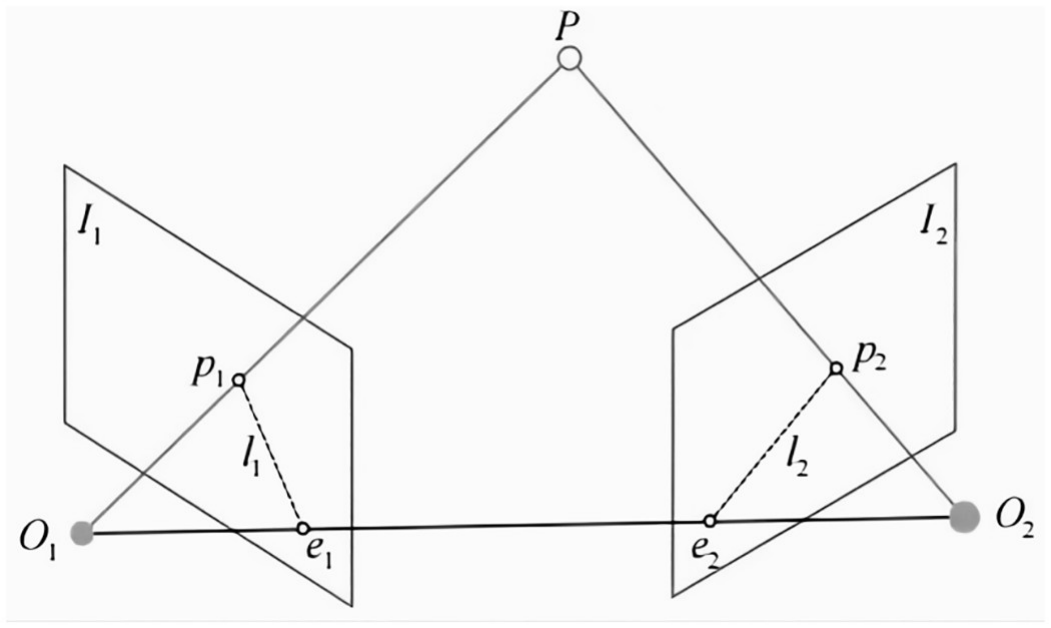
Geometric constraints on the poles.

The motion estimation session utilizes the matched pairs of feature points to calculate the relative motion between adjacent frames to construct the camera trajectory and the 3D structure of the scene. In this paper, a monocular camera based on the pinhole model is used for motion estimation using the 2D-2D pair of poles geometry method. The pair-polar geometry method estimates the camera motion using poles, which are the intersections of two polar lines in a pair of stereo images corresponding to the same 3D spatial point, as shown in Fig 12. In Fig 12, P is a point in three-dimensional space, *I*_1_,*I*_2_ is the two imaging planes,*O*_1_,*O*_2_ is the center of the two cameras, and *p*_1_,*p*_2_ is the projection of the P point on *I*_1_,*I*_2_ respectively. Connecting *O*_1_ *O*_2_, the intersection of the line with *I*_1_,*I*_2_ is *e*_1_ and *e*_2_ respectively.

In visual SLAM, the key to motion estimation is to solve the position transformation between two cameras by matching the feature points of the camera images. This process involves computing the rotation matrix of the right camera with respect to the left camera ***R*** and the translation vector as *t*.

Let *P* = [*X*,*Y*,*Z*]^*r*^, then the expression for the spatial coordinates of the point *p*_1_,*p*_2_ is:

$$p_{1}=KP$$ (18)

$$p_{2}=K(RP+t)$$ (19)

Then, eliminating the value of P in Eq:

$$K^{-1}p_{2}=RK^{-1}p_{1}+t$$ (20)

Simultaneous left-multiplication of the equation left and right by ${p_{2}}^{T}K^{-T}t^{\wedge }$, and approximate removal of the zero term, gives:

$$p_{2}^{T}K^{-T}t^{\wedge }RK^{-1}p_{1}=0$$ (21)

Set ***F*** = ***K***^−*T*^ ***t***^∧^ *R****K***^−1^) and there:

$$p_{2}^{T}Fp_{1}=0$$ (22)

Eqs (5)–(11) and (5)–(12) both describe the constraints on the polar geometry, which provide the basis for the camera pose estimation problem. Specifically, the estimation of camera pose can be divided into the following two steps: firstly, using the position data of the matched feature point *p*_1_,*p*_2_, the corresponding matrix ***F*** is computed; secondly, based on ***F***, the rotation matrix ***R*** and the translation vector *t* between the two frames are further solved.

Thus, the problem of motion estimation between two images is essentially transformed into a problem of solving the matrix.

1. Solve the matrix ***F*** based on the position of the matched feature point *p*_1_,*p*_2_;
2. Solve for the rotation matrix ***R*** and the translation vector *t* between the two frames based on the matrix ***F***.

Thus, the problem of motion estimation between two images is essentially transformed into a problem of solving the matrix.

In practical applications of visual SLAM, the visual odometry at the front end of the system processes only two consecutive frames of images. However, due to the unavoidable noise and computational errors in the images, the camera position estimation obtained from the visual odometry is often inaccurate, which leads to significant drift errors during prolonged motion. In order to minimize this error and ensure high localization and map building accuracy during continuous operation, the system needs to introduce loop closure detection and back-end optimization mechanisms. loop closure detection is used to identify whether there is a closed loop in the trajectory, while back-end optimization performs trajectory correction based on the loopback information. In addition, the back-end can use the real-time position data provided by the visual odometer for fine-tuning and re-positioning in combination with the loop closure detection results, so as to correct the drift error and obtain a more accurate motion trajectory.

Common algorithms for back-end optimization include the extended Kalman filtering method based on filters and the beam leveling method (Bundle Adjustment, BA) based on nonlinear optimization. In this study, the beam leveling method is used to optimize the estimated camera position and 3D structure by minimizing the reprojection error, where the reprojection error refers to the difference between the position predicted based on the visual odometry and the true position determined based on loop closure detection, which is usually expressed as:

$$\xi =\sum_{i=1}^{N}(x_{i}^{2}-z_{i}^{2})$$ (23)

where *N* is the number of feature points in the image and *x*_*i*_,*z*_*i*_ denotes the true and predicted positions of the ith feature point, respectively. The optimization objective, which is to minimize this error, defines the problem as a problem of solving nonlinear least squares.

The loop closure detection part uses an algorithm based on a self-encoder network, and to avoid wasting resources, the system sets the interval of loop closure detection to every 10 frames. That is, once the loopback is detected, the system will pause the loop closure detection in the next 10 frames and start again in the 11th frame.

The map construction of visual SLAM is divided into two parts: first, the scene map is constructed in real time based on the localization data from the front-end visual odometer; second, the scene map is updated based on the loop closure detection and back-end optimization results. There are various types of maps, including sparse maps, semi-dense maps, dense maps, raster maps, octree maps, 3D reconstruction maps, and topological maps, etc. The selection depends on the functional requirements of the visual SLAM system, such as dense maps for navigation and obstacle avoidance, 3D reconstruction maps for scene reproduction, and raster maps and topological maps for path planning. The visual SLAM system implemented in this study uses a semi-dense map approach similar to ORB-SLAM2.

### 4.2 Indoor visual SLAM system test experiments

#### 4.2.1 Experimental design

The hardware configuration for this experiment includes a high-performance computing platform equipped with a six-core Intel Core i5-9400F @ 2.90GHz CPU, 16GB of RAM, and an Nvidia GeForce GTX 1050 Ti 4GB GPU. This setup ensures the capability to process large-scale datasets and perform real-time computations. The image sensor used in the experiment is an 8MP RGB camera with the IMX219 model, capable of capturing 1080p video at 30 frames per second. The camera is connected to the computing platform via a USB interface, ensuring stable data transmission. The software environment consists of a system running the 64-bit Ubuntu 16.04 LTS operating system, with development environments including Matlab 2020 and C++ 14. The SLAM framework employed is ORB-SLAM2, an open-source visual SLAM solution capable of real-time pose estimation and map reconstruction in complex environments. Additionally, ROS (Robot Operating System) is utilized for managing data streams and integrating sensor data, while Python is used for data analysis and visualization of results.

The visual odometry in this experiment employs a feature-point-based approach, automatically extracting 1000 ORB feature points from each frame. These feature points are used for tracking and map construction. The backend optimization algorithm is the bundle adjustment method, which optimizes the estimated camera poses and 3D structures by minimizing the reprojection error. The similarity threshold for loop closure detection is set at 0.7 to reduce false positives and enhance detection accuracy, with a loop closure detection delay of 10 frames to improve the system’s real-time performance. This study focuses on the evaluation of loop closure detection algorithms using three selected datasets, which are utilized to assess the performance of the proposed method. The integration of ROS facilitates efficient data flow management and sensor data fusion, while Python supports comprehensive data analysis and result visualization.

In a complete visual SLAM system, the evaluation is not limited to comparing the precision and recall of individual algorithms but rather focuses on assessing the overall system performance. This is typically done through metrics such as trajectory error and average frame rate. Trajectory error, defined as the deviation between the estimated camera position calculated by the system and the true position, serves as a key indicator of the algorithm’s accuracy and trajectory consistency. The true position can be obtained through sensors like GPS or IMU, or manually calibrated. Trajectory error is commonly measured using Absolute Trajectory Error (ATE) and Root Mean Squared Error (RMSE). These metrics evaluate the system by comparing the estimated camera positions with the actual positions (which may be acquired through GPS, IMU, or manual annotation). ATE provides a measure of the positional error for individual frames, while RMSE offers an assessment of overall trajectory accuracy, reflecting the system’s position precision over long-distance movements. The calculation methods are as follows:

1. Obtain the estimated position of the camera from the visual odometer;
2. Align the estimated position with the true position on the ground;
3. Calculate the absolute error between the estimated position and the true position, which is used to express the position error for each frame:
$$ATE(i)=\begin{array}{c} \left\|x(i)-z(i)\right\| \end{array}$$ (24)
  where *x*(*i*), *z*(*i*) denote the real position and estimated position of the camera at frame i, respectively;
4. The RMSE is calculated by averaging the sum of squares of all errors and then squaring them to reflect the accuracy of the overall trajectory:
$$RMSE=\sqrt{\frac{1}{N}\sum_{i=1}^{N}ATE{\left(i\right)}^{2}}$$ (25)
  where N is the total number of frames in the trajectory.

Another evaluation metric is the average frame rate (Frames per Second, FPS), which expresses the number of frames processed per unit of time with the following formula:

$$FPS=\frac{N}{T}$$ (26)

where N denotes the total number of frames and T denotes the total time consumed.

The average frame rate measures the number of frames processed by the system per unit of time, serving as a critical indicator of the real-time performance of a SLAM system. A higher average frame rate indicates a stronger capability of the system to process images in real-time and maintain smooth operation. During the operation of a visual SLAM system, to ensure efficient real-time processing, the real-time frame rate is typically maintained at 70% to 80% of the average frame rate.

#### 4.2.2 Experimental results

This study compares the loop closure detection performance of our proposed method with that of ORB-SLAM2 across three datasets. The comparison focuses on the number of correct and incorrect correspondences in loop closure detection, with our method demonstrating superior precision. Detailed results are presented in Table 6.

**Table 6:** Statistics of loopback test results.

	Methodology of this study			ORB-SLAM2		
	correct amount	amount of error	accuracy	correct amount	amount of error	accuracy
TUM_fr3_loh	24	12	66.67%	20	15	57.14%
Lip6 Indoor	24	9	72.72%	18	14	56.25%
Bicocca Indoor	36	9	80.00%	25	16	60.98%

As shown in Table 6, our proposed method outperforms ORB-SLAM2 in terms of precision, based on the number of correct and incorrect correspondences in loop closure detection. In the TUM_fr3_loh dataset, despite ORB-SLAM2’s adaptability to environmental complexity and dynamic changes, our method achieves nearly 10 percentage points higher precision. For the Lip6 Indoor and Bicocca Indoor datasets, our method surpasses ORB-SLAM2 by 16.47% and 19.02% in precision, respectively. Overall, across the three datasets, the average precision of our method is 73.13%, significantly higher than the 58.12% achieved by ORB-SLAM2.

After conducting three experiments and counting the maximum, minimum, median and root mean square error of the absolute trajectory error, the final results are shown in Table 7.

**Table 7:** Statistics of loopback test results.

trajectory error	Methodology of this study				ORB-SLAM2			
	Max.	Min	Median	RMSE	Max.	Min	Median	RMSE
TUM_fr3_loh	0.1352	0.0451	0.0853	0.0887	0.1504	0.0506	0.1009	0.0952
Lip6 Indoor	0.1178	0.0347	0.0764	0.0732	0.1681	0.0517	0.1162	0.1234
Bicocca Indoor	0.1342	0.0342	0.0791	0.0816	0.1842	0.0682	0.1234	0.1341

As shown in Table 7, the results demonstrate that our proposed method consistently achieves lower trajectory errors across all datasets, with particularly significant improvements in the Bicocca Indoor dataset. In the TUM_fr3_loh dataset, the RMSE (Root Mean Squared Error) of trajectory error for our method is 0.0887 m, outperforming the 0.0952 m achieved by ORB-SLAM2. For the Lip6 Indoor dataset, our method’s trajectory error RMSE is 0.0732 m, markedly better than ORB-SLAM2’s 0.1234 m. In the Bicocca Indoor dataset, our method also shows superior performance with a trajectory error RMSE of 0.0816 m, compared to ORB-SLAM2’s 0.1341 m. The visual SLAM system implemented in this study consistently outperforms the ORB-SLAM2 algorithm in terms of trajectory error, with RMSE values around 0.08 m—approximately 0.05 m lower than those of ORB-SLAM2. This indicates that our system provides higher localization accuracy and greater stability.

Three experiments were conducted to calculate the average frame rate for each method and the final results are shown in Table 8.

**Table 8:** Comparison of average frame rates (frames/sec) for complete visual SLAM systems.

average frame rate	Methodology of this study	ORB-SLAM2
TUM_fr3_loh	15.52	14.81
Lip6 Indoor	16.41	20.18
Bicocca Indoor	16.96	19.62

As shown in Table 8, in the TUM_fr3_loh dataset, the average frame rate of our proposed method is 15.52 frames per second (fps), compared to 14.81 fps for ORB-SLAM2. Despite the complexity and variability of the environment in the TUM_fr3_loh dataset, our method’s slightly higher average frame rate indicates good processing efficiency and real-time performance. In the Lip6 Indoor dataset, the average frame rate of our method is 16.41 fps, while ORB-SLAM2 achieves 20.18 fps. In the Bicocca Indoor dataset, our method’s average frame rate is 16.96 fps, compared to 19.62 fps for ORB-SLAM2. Although our method’s average frame rate is slightly lower than ORB-SLAM2’s in the Lip6 Indoor and Bicocca Indoor datasets, it still exceeds the minimum required standard for real-time performance in all three datasets. Notably, in the TUM_fr3_loh dataset, our method demonstrates better processing speed than ORB-SLAM2, even in a complex environment, further proving its reliability in terms of real-time capability.

## 5. Discussion

This paper improves the loop closure detection algorithm in visual SLAM by incorporating deep learning techniques to enhance precise localization in high similarity scenes. The study primarily addresses two major challenges: the issue of mismatches in highly similar scenes and the sensitivity to minor visual changes. By introducing an improved feature extraction and matching mechanism, combined with real-time performance optimization techniques, the research significantly enhances the system’s localization accuracy and practicality.

In this study, we significantly improved the ORB-SLAM2 algorithm to address its inherent shortcomings, particularly the localization challenges in high similarity scenes. ORB-SLAM2 tends to experience mismatches in such environments due to its insufficient feature point matching mechanism, which struggles to differentiate between similar scene elements, leading to trajectory drift and inaccurate localization. To overcome these issues, we integrated deep learning techniques to optimize the feature extraction and matching process, greatly enhancing the system’s localization accuracy and robustness. By utilizing convolutional neural networks (CNNs), the system effectively learns and extracts high-level image features, which is crucial for improving scene differentiation. Additionally, deep learning models enable complex semantic analysis, helping to reduce mismatches in visually similar scenes. These advancements allow the SLAM system to maintain high localization accuracy and operational stability in various dynamic environments, especially in scenarios involving changes in lighting and seasonal transitions.

The improved algorithm demonstrated lower trajectory errors and higher localization accuracy on the Lip6 Indoor and Bicocca Indoor datasets. The study found that in the Lip6 Indoor dataset, the proposed method achieved an average accuracy of 73.13%, compared to 58.12% for the ORB-SLAM2 method. Furthermore, the trajectory error RMSE of the proposed method was significantly lower than that of ORB-SLAM2, reaching 0.0732m in the Lip6 Indoor dataset and 0.0816m in the Bicocca Indoor dataset. Although the frame processing rate of our algorithm was slightly lower than ORB-SLAM2, its accuracy was significantly improved, indicating substantial overall performance gains in highly similar scenes. These results validate the effectiveness and potential of deep learning techniques in enhancing SLAM system performance. Through these improvements, this study not only strengthened the localization accuracy of visual SLAM systems in highly similar indoor environments but also provided important technical references and experimental evidence for similar applications.

Current research in the field is also exploring the use of deep learning to enhance the performance of visual SLAM. Li et al. (2021) developed a real-time visual SLAM system based on deep learning, highlighting the broad potential of deep learning techniques to improve SLAM system performance [22]. Through specific algorithm optimizations and experimental validation, this study demonstrates the dual advantages of deep learning-enhanced visual SLAM systems in terms of accuracy and real-time performance in highly similar indoor environments. In summary, the contributions of this study are as follows: 1) A novel visual SLAM algorithm combined with loop closure detection is proposed to address the challenge of precise localization in high similarity scenes; 2) Improvements in the feature extraction and matching mechanism were achieved, effectively reducing mismatches and improving localization accuracy; 3) Through comparison with existing technologies such as ORB-SLAM2, the superiority of the proposed method in terms of practicality and efficiency has been demonstrated.

In this study, we successfully applied deep learning techniques to loop closure detection in visual SLAM, significantly improving the system’s localization accuracy and robustness in high similarity scenes. However, despite the positive outcomes, there are several limitations, which point to potential areas for future improvements and research. First, visual SLAM systems rely heavily on sensor inputs, such as cameras and motion sensors, where sensor noise and drift can lead to accumulated pose estimation errors, especially during long-term operations. Future research could explore more robust pose estimation algorithms or incorporate periodic global optimization methods, such as global map relocalization, to mitigate the impact of sensor error accumulation. Second, the adaptability of the model to highly dynamic environments needs further enhancement. While the current model performs well in static or slightly dynamic environments, its performance may degrade in scenarios with dense crowds or frequent object movement. Future studies should consider integrating online learning or incremental learning techniques to dynamically adjust and optimize the model in real time, enabling it to adapt to rapid environmental changes.Lastly, multimodal data fusion presents a promising direction for exploration. Although the proposed algorithm has significantly improved the accuracy of loop closure detection, in certain scenarios—such as long corridors or symmetric structures—the high similarity of visual features may still cause mismatches, leading to trajectory drift. Future research could address this by leveraging multimodal fusion techniques, combining data from other sensors, such as LiDAR or IMU, with visual information to enhance scene differentiation and reduce the risk of mismatches in highly similar environments.

## 6. Conclusion

The study improves the loop closure detection algorithm in visual SLAM systems by introducing deep learning techniques, focusing on addressing the challenge of accurate localization in highly similar indoor environments. The research findings are as follows:

1. In the multi-model comparison of Precision-Recall (P-R) curves, the area under the P-R curves of the proposed algorithm exceeded 0.83 across all three datasets, outperforming traditional algorithms such as ORB-SLAM2, GeM, and NetVLAD. The average precision of the proposed algorithm reached 73.13%, compared to only 58.12% for ORB-SLAM2. Notably, in the Bicocca Indoor dataset, the proposed method achieved an impressive precision of 80.00%, while ORB-SLAM2 only reached 60.98%, indicating a significant improvement in localization accuracy in complex and high similarity scenes.
2. In experimental tests, the proposed algorithm achieved root mean square errors (RMSE) of 0.0887m, 0.0732m, and 0.0816m in the TUM_fr3_loh, Lip6 Indoor, and Bicocca Indoor datasets, respectively, significantly lower than the ORB-SLAM2 algorithm’s RMSEs of 0.0952m, 0.1234m, and 0.1341m. This demonstrates the algorithm’s superior localization accuracy in high similarity scenes.
3. Compared to the traditional ORB-SLAM2 method, the proposed algorithm significantly reduced the occurrence of mismatches by optimizing the feature extraction and matching mechanisms, ensuring reliable system localization, particularly in visually similar and complex environments. Although the frame rate of the proposed algorithm was slightly lower than that of ORB-SLAM2–16.41 frames per second and 16.96 frames per second on the Lip6 Indoor and Bicocca Indoor datasets, respectively—it still met the requirements of real-time SLAM systems. The proposed method not only enhances localization accuracy and precision but also maintains good real-time performance.

Although this study achieved significant results in highly similar indoor environments, its adaptability to dynamic environments still requires improvement. Future work will focus on the impact of factors such as lighting variations and small object movements on feature stability and matching accuracy to enhance adaptability to complex environments and reduce reliance on specific types of features. Comparisons with existing techniques have demonstrated the superiority and practicality of the proposed method. Despite some limitations, this study identifies clear directions for future improvements, laying the groundwork for the further development of visual SLAM technology in similarly challenging applications.

## Supporting information

S1 Data (ZIP)
